# Prevalence of Insomnia and Factors Influencing Its Incidence in Students of Tbilisi State Medical University: A Cross-Sectional Study

**DOI:** 10.7759/cureus.46084

**Published:** 2023-09-27

**Authors:** Siddhant Solanki, Arun Venkiteswaran, Prithvi Saravanabawan

**Affiliations:** 1 Neuropsychiatry, California Institute of Behavioral Neurosciences & Psychology, Fairfield, USA; 2 Medicine, Tbilisi State Medical University, Tbilisi, GEO; 3 Neurology, Tbilisi State Medical University, Tbilisi, GEO

**Keywords:** sleep patterns, cross-sectional study, onset insomnia, social media usage, academic performances, sleep hygiene, mental health issue, excessive workload, medical students, insomnia

## Abstract

Sleep is essential for overall health and well-being, however, medical students often face challenges in the form of insomnia or sleep-related disorders. This cross-sectional study investigates the factors influencing the incidence of insomnia in medical students at Tbilisi State Medical University during the 2022-2023 academic year. Using an online questionnaire, data was collected from 174 respondents with self-reported insomnia, and processed with spreadsheet software. The questionnaire collected demographic information, and data regarding sleeping patterns, and asked respondents to choose ‘yes’ or ‘no’ for each factor and its effect on their self-reported insomnia. After applying inclusion and exclusion factors, 122 responses were used for analysis. A chi-square analysis was conducted to verify the statistical significance of the data (p = 0.002002). The study revealed a high prevalence of 70.11% of respondents reporting sleeping difficulties. A total of 71.30% of respondents reported excessive workload as the most significant factor contributing to the incidence of their insomnia. Sixty-eight percent of respondents reported mental health issues (including anxiety and depression), and 65.5% reported improper sleep hygiene (including daytime napping and irregular sleep schedules) to play a role in the incidence of their insomnia. Social media and entertainment platform usage (59.8%) and stimulant consumption (48.4%) were comparatively less prominent but still noteworthy contributors to insomnia. The study also found that the majority of respondents (59.8%) experience onset-related insomnia, while 40.4% experience maintenance-related insomnia. This study found excessive workload to be the factor that most influenced the incidence of insomnia in medical students at Tbilisi State Medical University. This can be attributed to the increased academic load a medical student has to face and the worry about academic performance. Proper sleep hygiene, mental health support, and workload adjustments are suggested to decrease the incidence of insomnia among medical students at Tbilisi State Medical University.

## Introduction

Sleep is essential for normal physiological functioning and the good health of the human body, and a lack of sleep can manifest as increased stress, mood disorders, performance deficits, and reduced quality of life [1]. Medical students are no exception to this, they are especially vulnerable to poor sleep and sleep-related disorders [2]. Given the damaging effects of lack of sleep, knowledge of the factors that surround it can help them make decisions that will improve their general well-being and academic performance.

Improper sleep can be categorized into seven major forms, with insomnia being the most common. According to the International Classification of Sleep Disorders - Third Edition, insomnia is understood to be a disorder of sleep initiation or maintenance that, despite an adequate environment and opportunity, results in daytime consequences [3].

The lack of a good night’s sleep is known to cause anxiety, change in mood, and poor performance. It’s also understood that insomnia and mental health issues share a bidirectional relationship [4]. With medical students more likely to be impacted by mental health issues, there are high chances for their sleep to be impacted [2]. This increases the incidence of insomnia, which was seen during the COVID-19 pandemic [5]. This also leads to a vicious situation where students may choose to combat daytime sleepiness with stimulants such as caffeine further worsening their sleep cycle [6]. Excessive stimulant intake, such as caffeine from coffee or energy drinks can lead to further development of insomnia [7].

Improper sleep hygiene such as daytime sleeping can also contribute to sleeping difficulties [8]. This is observed among students choosing to take daytime ‘naps’ to get rested after an improper night’s sleep, which further damages their sleep schedules, and increases the incidence of insomnia. The use of social media, entertainment platforms, and looking at screens for an extended period of time, has also been shown to cause sleeping difficulties [9].

Studies examining the incidence of sleeping issues in medical students also highlight increased academic pressure, examinations, late-night internet usage, mental health problems, and excessive daytime sleepiness [2,10]. The factors influencing the incidence of insomnia can be broadly classified into five main factors, i.e. (i) mental health issues, (ii) stimulant consumption, (iii) improper sleep hygiene, (iv) use of social media and other entertainment platforms, and (v) excessive workload. This study aims to investigate the level to which these five factors listed above impact the incidence of insomnia in medical students at Tbilisi State Medical University, during the 2022-2023 academic year.

## Materials and methods

Study design

In this study, a cross-sectional study design was used, to determine the factors that influence the prevalence and incidence of insomnia among medical students in Tbilisi State Medical University. The study was conducted with a three-part questionnaire. The first section collected demographic information. The second section asked respondents to gauge the impact that self-declared insomnia had on their lives. The third section presented respondents with five factors, and participants were asked to rate ‘yes’ or ‘no’ for each factor if they believed that it contributed to the incidence of their self-reported insomnia. To access the second and third sections, respondents had to answer ‘Yes’ to the question ‘Do you think you have chronic sleeping difficulty (insomnia) at night?’

Data collection

Data was collected through the convenience sampling method, by distributing the questionnaire online with an online query generator software. The questionnaire was disseminated through contacts and university distribution mechanisms such as batch WhatsApp groups and emails. Effort was made to ensure that the data collected was as diverse as possible.

Demographic information collected through the questionnaire consisted of name, sex, year of study, and age. To verify the response, respondents were asked to share their name, which was then discarded and separated from the data once authenticity had been established. Following this, all personal identifiers were removed, and data was anonymized before statistical analysis.

Sampling method

A total of 174 responses were received, and accounting for the inclusion and exclusion criteria, 122 responses were deemed valid and used for analysis. The inclusion criteria were as follows: (a) the participant had to be an active student of Tbilisi State Medical University, (b) the participant had self-reported having insomnia at night. The exclusion criteria were as follows: (a) anyone who is not an active medical student at Tbilisi State Medical University. (b) Individuals with a history of diagnosed sleep disorders other than insomnia.

Participant consent and information

Prior to starting the questionnaire, participants were asked for their explicit informed consent regarding the collection and management of the data collected during the study. The amended Helsinki Declaration was followed during the study's execution. Participants were not paid for their involvement in the study, and there were no associated expenditures.

Statistical analysis

Statistical analysis was done using online resources (socscistatistics.com) and (quantpsy.org). Graphs and tables were created with online data management software. Pearson chi-square tests were used and the level of statistical significance was set at P<0.05.

The p-value calculated for this study measured at (p < 0.002002) suggesting a 0.2% chance that the relationship between the factors taken in this study (mental health, stimulant consumption, improper sleep hygiene, social media, workload) and insomnia is coincidental, therefore rejecting the null hypothesis (factors show no relevance to insomnia), in favor of the alternative hypothesis (factors show relevance to insomnia).

Prevalence was calculated using the formula ((Number of self-reported cases in the study/number of persons in the study population) x 100). The study population was taken to be the total number of respondents at 174, and the number of self-reported cases in the study was 122, yielding a prevalence of 70.11%.

Additionally, chi-square analysis for p values was conducted between individual factors such as social media and workload (p=0.00001), mental health, and stimulant consumption (p=0.003473); both of which produced a result of below p < 0.05 strongly suggesting that the factors are empirically related to each other in favor of the alternate hypothesis.

## Results

Participant characteristics

Among the 122 responses that fit the inclusion and exclusion criteria, 57 were male (46.7%) and 65 were female (53.3%). Distribution amongst the years of education was First Year - 15 (12.30%), Second Year - 37 (30.30%), Third Year - 19 (15.60%), Fourth Year - 17 (13.90%), Fifth Year - 12 (9.80%), Sixth Year - 22 (18%). The youngest respondent was 17 years old and the oldest respondent was 28, and the average age of the respondents was 21 years old. Table 1 presents the various participant characteristics.

**Table 1:** Illustration of participants’ gender, academic year, attribution to insomnia, satisfaction with current sleeping pattern, and hours slept per day in a normal week of university.

Total respondents:	174	
Used responses:	122	
Sex:		
Male	57	46.70%
Female	65	53.30%
Distribution amongst academic years		
1st year	15	12.30%
2nd year	37	30.30%
3rd year	19	15.60%
4th year	17	13.90%
5th year	12	9.80%
6th year	22	18.00%
Attribution of insomnia		
Onset related	73	59.80%
Maintenance-related	49	40.20%
Satisfaction with current sleeping pattern		
Very satisfied	3	2.50%
A little satisfied	14	11.50%
Neutral	28	23.00%
A little dissatisfied	39	32.00%
Very dissatisfied	38	31.10%
Hours slept per day in a normal week of university:		
Hours	Count	Percentage
1	1	0.57%
2	2	1.15%
3	10	5.75%
4	23	13.22%
5	21	12.07%
6	40	22.99%
7	18	10.34%
8	9	5.17%

Onset or maintenance

The study focused on two types of insomnia, and respondents were asked to choose if they experienced difficulty falling asleep (onset-related insomnia) or maintaining sleep (maintenance-related insomnia). The majority of the respondents (n=73, 59.8%) reported that they experienced difficulty falling asleep while (n=49, 40.2%) of the respondents reported difficulty maintaining their sleep.

Sleep satisfaction and impact on life

Participants reported their satisfaction with current sleeping patterns as follows: Very satisfied - Three (2.50%), A little satisfied - 14 (11.50%), Neutral - 28 (23%), A little dissatisfied - 39 (32%), Very dissatisfied - 38 (31.1%). The majority of participants (63.1%) were a little or very dissatisfied with their current sleeping patterns. Table 2 shows the impact of self-reported insomnia on the lives of participants.

**Table 2:** Illustration of the impact of self-reported insomnia on the lives of participants.

Rating	Impact on learning (frequency)	Aggregate (learning)	Impact on social life (frequency)	Aggregate (social life)
1	2	20	1	21
2	6		10	
3	12		10	
4	17	73	18	69
5	16		19	
6	17		12	
7	23		20	
8	14	29	16	32
9	9		10	
10	6		6	

Impact on Learning

Respondents were asked to rate the impact of their self-reported insomnia on their learning, with one indicating minimum impact and 10 indicating maximum impact. The data was aggregated and then divided into three divisions. These divisions were minimal impact (from one to three) which 16.39% (n=20) participants selected, moderate impact (from four to seven) which 59.83% (n=73) participants selected, and severe impact (from eight to 10) which 23.77% (n=29) participants selected. The majority of the participants (n=23) selected a rating of seven.

Impact on Social Life

Respondents were asked to rate the impact of their self-reported insomnia on their social life, with one indicating minimum impact and 10 indicating maximum impact. The data was aggregated and then divided into three divisions. These divisions were minimal impact (from one to three) which 17.21% (n=21) participants selected, moderate impact (from four to seven) which 56.55% (n=69) participants selected, and severe impact (from eight to 10) which 26.22% (n=32) participants selected. The majority of the participants (n=20) selected a rating of seven.

Mental health issues

When asked to report if mental health factors played a role in the incidence of their self-reported insomnia, n=83 (68%) of participants said yes, while n=39 (32%) said no. Anxiety and depression were mentioned as examples of mental health factors, however, the specifics of which mental health issue the respondent may be suffering from, or the status of their diagnosis was not collected. Table 3 provides information about the factors influencing the incidence of insomnia.

**Table 3:** Illustration of the factors influencing the incidence of insomnia.

Factors	YES	Percentage	NO	Percentage
Mental health	83	68%	39	32%
Stimulant consumption	59	48.40%	63	51.60%
Improper sleep hygiene	80	65.60%	42	34.40%
Social media	73	59.80%	49	40.20%
Excessive workload	87	71.30%	35	28.70%

Stimulant consumption

When asked to report if stimulant consumption played a role in the incidence of their self-reported insomnia, n=59 (48.4%) of participants said yes, while a majority n=63 (51.6%) said no. Specific data on which stimulants, such as coffee, or energy drinks, nor the time and frequency of consumption were collected.

Improper sleep hygiene

Sleep hygiene is a multifactorial component and includes a wide spectrum of daily habits and constructs, including daytime sleeping, irregular sleep schedule, etc. When asked to report if improper sleep hygiene played a role in the incidence of their self-reported insomnia, a majority (n=80, 65.6%) of participants said yes, while n=42 (34.4%) said no.

Use of social media and other entertainment platforms

When asked to report if use of social media and other entertainment platforms played a role in the incidence of their self-reported insomnia, n=73 (59.8%) of participants said yes, while n=49 (40.2%) said no. Detailed data about time of use, and the hours spent on the platforms were not collected. Instagram and Netflix were used as examples of such social media and entertainment platforms.

Excessive workload

**Figure 1:**
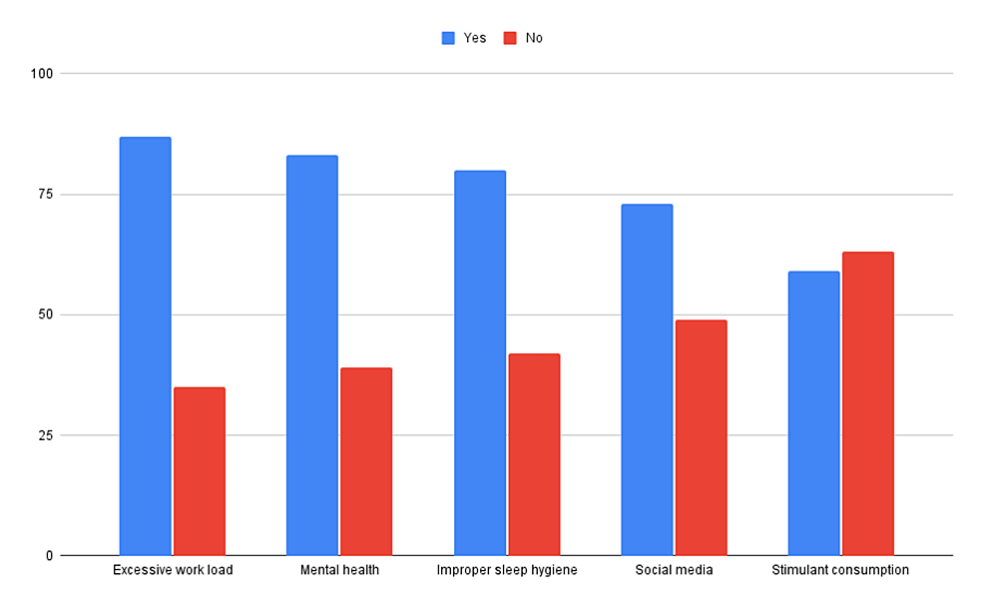
The collective responses of participants for each factor that influences the incidence of insomnia.

When asked to report if an excessive workload played a role in the incidence of their self-reported insomnia, a significant majority n=87 (71.3%) of participants said yes, while n=35 (28.7%) said no. Excessive workload is one of the most attributed components to self-reported insomnia among medical students in accordance with the data collected for this study. Figure 1 shows the collective responses of participants for each factor that influences the incidence of insomnia.

## Discussion

In this cross-sectional study, we aimed to identify the factors influencing the incidence of insomnia, in medical students at Tbilisi State Medical University. University students are at higher risk of sleeping problems, and this is reflected in our data, with 70.11% of our respondents self-reporting insomnia [11].

The factor that a majority of our participants reported to have a major effect on their sleep was excessive workload (71.30%), followed by mental health (68%), and improper sleep hygiene (65%). Furthermore, the average reported duration of sleep on a normal day at university from our study was five hours and 18 minutes which is lower than that reported by the meta-analysis of 109 research papers at six hours 30 minutes hours [12].

With the average age of our respondents being 21, the majority of them are making the transition from adolescence to adulthood, which is a high-risk group for sleep problems [11]. When asked to choose if their sleeping difficulty was onset-related or maintenance-related, the majority of our respondents (59.8%) chose onset-related insomnia. Improper sleep hygiene which includes daytime sleeping is a major contributor to onset-related sleeping difficulties [8,13].

Mental health and related issues such as anxiety and depression have long been known to cause difficulties with sleeping [14]. With 68% of our respondents indicating that they believe they have mental health issues, the link between mental health and sleeping difficulties can be established.

Two of the lower-rated factors when it came to the incidence of self-reported insomnia as reported by our respondents were social media (59.8%) and stimulant consumption (48.4%). We can presume that social media as such may not cause insomnia, but can develop feelings of anxiety or ‘Fear of Missing Out’, and exposure to a screen may cause decreased melatonin secretion [15]. These are in turn factors that may lead to the incidence of insomnia.

Having conducted this study, we encourage medical students of Tbilisi State Medical University and medical students all over the world to give greater importance to the quality of their sleep. Proper sleep hygiene is the easiest to develop and could result in the improvement of self-reported insomnia. In the future, to better understand insomnia in medical students, additional studies can be conducted with bigger sample sizes, and with more detailed questionnaires to gain a deeper understanding of the factors influencing the incidence of insomnia and ways to mitigate them.

Limitations

Data collection was conducted through convenience sampling, so equal representation from all six years of the university was not available. Furthermore, the study did not collect specific details under each of the factors and did not use standardized scales for measuring insomnia or quality of sleep. Additionally, neither randomization nor systematic techniques were used in our sampling strategy. Nonetheless, it is important to emphasize that our findings are intriguing and have prompted us to pose novel research questions that merit further investigation.

## Conclusions

The study revealed that the prevalence of insomnia is 70.11% and excessive workload, mental health-related issues, and improper sleep hygiene are the major factors that influence the incidence of insomnia in medical students at Tbilisi State Medical University. The average sleep duration for medical students at Tbilisi State Medical University on a normal day of university is five hours and 18 minutes. A total of 59.8% of respondents reported difficulty falling asleep and had onset-related insomnia. Data also revealed that insomnia impacts the social life and learning of medical students at Tbilisi State Medical University. Medical students should take measures to improve hours of sleep, seek mental health support, and with support from the university administration adjust their workload to improve their insomnia.

## Appendices

**Table 4:** Survey questionnaire

SURVEY QUESTIONNAIRE		
Confidentiality disclaimer: Your answers to this survey are fully confidential and your personal information will not be published. Your name is required to verify the authenticity of the response, and will not be published. The conductors of the study will protect your information, so no one will be able to connect your responses and any other information that identifies you. This survey may contain questions that may be personal. You can exit and terminate your participation at any point before submission. Your electronically signed consent is necessary to move forward.		
Respondent information		
Questions	Instructions to the respondent	Options/Answers
Full name:	We require your name to establish the authenticity of the response. Data will be anonymised once the authenticity is verified.	Open-ended answer
Sex	-	Male/female
Year:	-	1st year/2nd year/3rd year/4th year/5th year/6th year
Age:	-	Open-ended answer
How many hours do you sleep per day in a normal week of university?	-	Open-ended answer
Do you think you have chronic sleeping difficulty (insomnia) at night?	According to the International Classification of Sleep Disorders - Third Edition, insomnia is understood to be a disorder of sleep initiation or maintenance that, despite an adequate environment and opportunity, results in daytime consequences. Please select YES if you fulfil these requirements	Yes/no
Impact of Insomnia		
How satisfied are you with your current sleep pattern?	-	Very satisfied/a little satisfied/neutral/a little dissatisfied/very dissatisfied
To what extent do you consider your insomnia to interfere with learning?	-	On a scale of 1-10, 1 being least impact, and 10 being greatest impact.
To what extent do you consider your insomnia to interfere with your personal (social) life?	-	On a scale of 1-10, 1 being least impact, and 10 being greatest impact.
How would you attribute the cause of your insomnia?	-	Onset-related (having trouble falling asleep)/Maintenance-related (having trouble maintaining the state of sleep)
Factors influencing the incidence of insomnia		
Do you believe that mental health issues could be the cause of your insomnia?	E.g.: anxiety, depression, etc	Yes/no
Do you feel excess stimulant consumption could be a cause of your insomnia?	E.g.: coffee, soft drinks, caffeine, etc	Yes/no
Do you believe that your insomnia may be related to poor sleep hygiene?	E.g.: daytime sleeping, irregular sleep schedule, etc.	Yes/no
Do you believe that your insomnia may be influenced by your use of social media and other entertainment platforms?	E.g: Netflix, Instagram, etc	Yes/no
Do you think your insomnia may be caused by having excessive work load?	-	Yes/no
